# Associations between Exercise-Induced Pulmonary Hemorrhage (EIPH) and Fitness Parameters Measured by Incremental Treadmill Test in Standardbred Racehorses

**DOI:** 10.3390/ani12040449

**Published:** 2022-02-12

**Authors:** Chiara Maria Lo Feudo, Luca Stucchi, Giovanni Stancari, Elena Alberti, Bianca Conturba, Enrica Zucca, Francesco Ferrucci

**Affiliations:** 1 Equine Sports Medicine Laboratory “Franco Tradati”, Department of Veterinary Medicine and Animal Sciences, Università Degli Studi di Milano, 26900 Lodi, Italy; chiara.lofeudo@unimi.it (C.M.L.F.); elena.alberti@unimi.it (E.A.); enrica.zucca@unimi.it (E.Z.); 2 Veterinary Teaching Hospital, Department of Veterinary Medicine and Animal Sciences, Università Degli Studi di Milano, 26900 Lodi, Italy; luca.stucchi@unimi.it (L.S.); giovanni.stancari@unimi.it (G.S.); bianca.conturba@unimi.it (B.C.)

**Keywords:** EIPH, horses, racehorses, standardbred, treadmill test, lactate, poor performance, sports medicine

## Abstract

**Simple Summary:**

Exercise-induced pulmonary hemorrhage (EIPH) frequently affects racehorses worldwide and has been widely associated with poor performance; however, scientific evidence supporting this observation is low. The present retrospective study aims to evaluate objectively whether the presence and grade of EIPH could affect some fitness parameters, measured during an incremental treadmill test, in poorly performing Standardbred racehorses. For this purpose, the association between EIPH and the results of a treadmill metabolic test (including blood lactate analysis and venous blood gas analysis) were evaluated in 81 Standardbred racehorses. No relationship between EIPH and aerobic/anaerobic capacity was observed, suggesting that EIPH may affect performance in a different manner. However, EIPH-affected horses were shown to reach higher hematocrit values during exercise compared to EIPH-negative horses; therefore, it may be hypothesized that hemoconcentration may take part in the pathogenesis of EIPH by increasing the pulmonary capillary pressure.

**Abstract:**

Exercise-induced pulmonary hemorrhage (EIPH) is a condition affecting up to 95% of racehorses, diagnosed by detecting blood in the trachea after exercise and/or the presence of hemosiderophages in the bronchoalveolar lavage fluid (BALf). Although EIPH is commonly associated with poor performance, scientific evidence is scarce. The athletic capacity of racehorses can be quantified through some parameters obtained during an incremental treadmill test; in particular, the speed at a heart rate of 200 bpm (V200), and the speed (VLa4) and the heart rate (HRLa4) at which the blood lactate concentration reaches 4 mmol/L are considered good fitness indicators. The present retrospective study aims to evaluate whether EIPH could influence fitness parameters in poorly performing Standardbreds. For this purpose, data from 81 patients regarding their V200, VLa4, HRLa4, peak lactate, maximum speed, minimum pH, and maximum hematocrit were reviewed; EIPH scores were assigned based on tracheobronchoscopy and BALf cytology. The association between the fitness parameters and EIPH was evaluated through Spearman’s correlation analysis. No relationship between EIPH and V200, VLa4, and HRLa4 was observed. Interestingly, EIPH-positive horses showed higher hematocrit values (*p* = 0.0072, r = 0.47), suggesting the possible influence of the hemoconcentration on the increase of pulmonary capillary pressure as a part of the pathogenesis of EIPH.

## 1. Introduction

Exercise-induced pulmonary hemorrhage (EIPH), defined as bleeding occurring from the lungs during exercise, is a highly frequent condition among racehorses worldwide [1], affecting from 43% to 75% of racehorses when diagnosed on a single examination, and up to 95% in the case of repeated examinations [2]. Its diagnosis is based on the detection of blood in the trachea from 30 to 120 min after strenuous exercise [3], and/or the presence in the bronchoalveolar lavage fluid (BALf) of red blood cells, acutely, or hemosiderophages in subacute and chronic cases [4]. EIPH has frequently been associated with poor performance in Thoroughbreds and Standardbreds [5,6,7,8,9]; however, scientific evidence supports this hypothesis only partially. Different authors reported an association between the presence of EIPH and the likelihood of a lower position in races [7,10,11]. In particular, in a study, horses with EIPH of grade ≥ 2 showed to have lower odds of finishing in the first three positions, and horses with EIPH of grade ≥ 1 finished at a longer distance behind the winner compared to EIPH-negative horses [7]; also, in another study, EIPH-positive horses tended to finish unplaced [11]. Moreover, severe forms of EIPH (grade 4) have been associated with a shorter duration of the racing career [9], and EIPH of grade ≥ 2 has been associated with a lower likelihood to be in the 90th higher percentile for race earnings [7]. In contrast, other authors failed to detect any influence of EIPH on the finishing position of racehorses [12,13,14,15,16], nor on the finishing time in a race [15]. However, to date, no studies have managed to quantify objectively the lower performance of EIPH-affected horses compared to EIPH-negative horses. To assess in a quantitative way the athletic capacity of the horses, a standardized incremental exercise test on a treadmill may be performed. The maximal oxygen consumption (VO_2max_) reflects the maximal aerobic capacity and is considered as a good predictor of athletic performance [17]. Moreover, the measurement of the blood lactate concentration can provide useful information on both the aerobic and anaerobic capacities of the horse; the velocity at which the horse’s blood lactate concentration reaches 4 mmol/L (VLa4) represents the aerobic–anaerobic threshold and is a good indicator of aerobic capacity [18]. High values of VLa4 have been associated with superior performance in different studies [19,20,21]. An indication of anaerobic capacity could be provided by the measurement of the post-exercise peak lactate levels [22], and higher concentrations are reported to be found in faster horses [23,24] with better performance [25]; however, the lactate concentration is affected by many variables, including the rates of flux between fluid compartments. Therefore the use of the peak lactate concentrations as an indicator of anaerobic capacity appears limited [26]. The present study aims to evaluate whether the presence and grade of EIPH could have an influence on selected performance parameters measured during a standardized incremental treadmill test in poorly performing Standardbred horses.

## 2. Materials and Methods

### 2.1. Horses

The clinical records from a population of Standardbred racehorses referred to the Equine Medicine Unit of the Veterinary Teaching Hospital of the University of Milan (Italy) for poor performance evaluation between 2002 and 2020 were retrospectively reviewed. All horses were in full training and underwent a complete diagnostic protocol, including the collection of history, clinical physical examination, laboratory analysis (complete blood count, blood chemistry, and arterial blood gas analysis), lameness evaluation, electrocardiogram, thoracic ultrasonography, upper airways endoscopy at rest, incremental high-speed treadmill metabolic test (plasma lactate analysis and Holter registration during exercise), dynamic endoscopy of the upper airways on a high-speed treadmill, tracheobronchoscopy 30 min after dynamic endoscopy, and BALf collection, including its cytological examination, performed 24 h after the dynamic endoscopy on the treadmill.

Horses showing signs of systemic illness, lameness, cardiac murmurs of grade > 3/6 [27], clinically significant valvular regurgitations [28], clinically significant cardiac arrhythmias [29,30], dynamic upper-airway obstructions (DUAOs), or rhabdomyolysis (stiffness, reluctance to move, sweating, tachypnea, tachycardia, myoglobinuria, and/or creatin-kinase > 735 U/l at 6 h after exercise [31]) were excluded from the study, since these afflictions could influence athletic performance.

### 2.2. Incremental Treadmill Test

Before performing the incremental treadmill test, the horses were conditioned to the high-speed treadmill (Sato I, Uppsala, Sweden) with two daily sessions. On the third day, the test was performed: the belt was inclined with a 5% slope, the protocol started with a warm-up of 4 min walk (1.5 m/s) and 3 min trot (6 m/s), followed by 1 min phases increasing the speed by 1 m/s until the horse was no longer able to maintain the treadmill speed; at the end of the test, the horses were walked for 30 min with a 0% slope to cool down [31].

Blood samples were collected during the test with the aid of a 14 G Teflon venous catheter placed in the left jugular vein and connected to an extension tube; blood samples were taken at rest, after the warm-up phase, at the end of each speed phase, and at 1, 5, 15, and 30 min during the cool down. To perform plasma lactate analysis, blood was transferred into tubes containing 10 mg sodium fluoride and 2 mg potassium oxalate for 1 mL of blood. The samples were centrifugated within 15 min and refrigerated, and the plasma lactate was measured with the enzymatic colorimetric method using a lactate dry-fast kit for the automatic system (Uni Fast System II Analyzer, Sclavo, Italy) and reagents supplied by the manufacturer [32]. In some horses, an aliquot of blood collected in heparinized syringes at each phase was used to measure the blood pH (48 horses) and hematocrit (31 horses) by means of a blood gas analyzer (Opti CCA, Opti Medical System, Roswell, NM, USA).

Throughout the duration of the treadmill test, the heart rate was monitored in real-time using a heart rate monitor (Polar, Equine Inzone FT1, Steinhausen, Switzerland); moreover, an ECG was obtained continuously before, during. and after exercise by means of a Holter recorder (Cardioline^®^ Click Holter, Trento, Italy) [30].

### 2.3. Fitness Parameters

Data about the values of plasma lactate, heart rate, and eventually pH and hematocrit at each speed phase and during the cool down were obtained at the end of the test and collected on an electronic sheet (Microsoft Excel, Redmond, WA, USA). The fitness parameters obtained from the treadmill test were:

- VLa4: speed at a plasma lactate concentration of 4 mmol/L;
- HRLa4: heart rate at a plasma lactate concentration of 4 mmol/L;
- V200: speed at a heart rate of 200 bpm;
- Peak lactate: maximum plasma lactate concentration reached during the treadmill test or cool down;
- V max: maximum speed reached during the test until fatigue;
- pH min: minimum pH reached during the test;
- Ht max: maximum hematocrit reached during the test.

The values of VLA4 and HRLa4 were calculated by means of specific software (Lactate-E 1.0) [33]; after entering the data about the plasma lactate concentration and heart rate collected during treadmill exercise at each speed, this software used inverse prediction to provide precise lactate threshold markers.

### 2.4. Airway Endoscopy

The day after the incremental test, the horses underwent a dynamic upper airway treadmill endoscopy at racing speed. After a warm-up of a 4 min walk (1.5 m/s) and 5 min trot (6 m/s) with a 5% slope, the treadmill was temporarily stopped. A videoendoscope (ETM PVG-325, Storz, Tuttlingen, Germany) was passed into the nasopharynx of the horse and held in position with straps. Then, the treadmill was rapidly accelerated up to maximal speed (speed at maximum heart rate) for a 1600–2100 m distance or until fatigue. Thirty minutes after the end of the exercise, tracheobronchoscopy was performed. The horses were restrained in a stock and contained with a twitch. A flexible videoendoscope (EC-530WL-P, Fujifilm, Tokyo, Japan) was passed through the left nostril, and the upper and lower tracts of the respiratory system were visualized. The eventual presence of blood in the trachea and the mainstem bronchi was graded from 0 to 4 based on a reported scoring system (tracheal blood score, TBS) [3].

### 2.5. BALf Collection and Cytological Examination

Twenty-four hours after the treadmill exercise, BALf was collected; with this aim, horses were restrained in a stock and sedated with detomidine hydrochloride (0.01 mg/kg IV; Domosedan; Vetoquinol, Italy). Airway endoscopy was performed as described above. To perform the BAL, 60 mL of a 0.5% lidocaine hydrochloride solution was sprayed at the level of the tracheal bifurcation in order to inhibit the coughing reflex; then, the endoscope was passed into the bronchial tree until it was wedged firmly within a segmental bronchus. Here, a 300 mL pre-warmed sterile saline 0.9% was instilled, and the fluid was immediately aspirated. The BALf sample was stored in sterile ethylenediaminetetraacetic acid (EDTA) tubes and processed within 90 min. To perform the cytological examination, a few drops of pooled BALf were cytocentrifugated (Rotofix 32, Hettich Cyto System, Tuttlingen, Germany) at 500 rpm for 5 min. The slides were air dried, stained with May-Grünwald Giemsa and Perl’s Prussian blue, and observed under a light microscope at 400× and 1000× for 400-cell leukocyte differential counting [34]. To evaluate EIPH, 100 macrophages were assessed. The percentage of hemosiderophages on the total of macrophages was calculated and hemosiderin was scored from 0 to 4 based on the grading of blue coloration in the cytoplasm of the macrophages [35]; then, the percentage of hemosiderophages was multiplied by the median hemosiderin score to obtain a simplified total hemosiderin score (sTHS), with a maximum score of 400 [36].

### 2.6. Statistical Analysis

All data were analyzed using descriptive statistics and evaluated for normality by means of the Shapiro–Wilk test. The influence of age and weight on every parameter was evaluated using the Spearman correlation, while the influence of sex was evaluated by means of the Kruskal–Wallis test (when considering stallions, geldings, and mares) and the Mann–Whitney test (when considering males and females). The association between the TBS and all the fitness parameters, the BALf leukocyte differential cell count, and the sTHS was evaluated using Spearman’s correlation. The same test was used to analyze the relationship between the sTHS with the fitness parameters and the BALf leukocyte differential cell count. The fitness parameters, the BALf leukocyte differential cell count, and the sTHS in the EIPH positive (TBS ≥ 1) and negative groups were compared by means of the Mann–Whitney test and the unpaired *t* test. The relationship between the BALf leukocyte differential cell count and the fitness parameters was evaluated by means of Spearman’s correlation. Since the percentage of neutrophils is related to some fitness parameters [32], a group of horses presenting a neutrophils percentage of < 5% [37] in the BALf was identified (Neu5 group); among them, the association of the different fitness parameters with age, weight, TBS, and sTHS was evaluated with Spearman’s correlation. The data are presented as the mean ± standard deviation (SD) if normally distributed, and as the median and interquartile range (IQR) if not normally distributed. Statistical significance was set at *p* < 0.05. The data were analyzed using a commercially available statistical software package (GraphPad Prism 9.1.0 for MacOS; GraphPad Software, San Diego, CA, USA).

## 3. Results

### 3.1. Study Population

Among the 230 Standardbred racehorses that underwent a complete diagnostic protocol for poor performance, 81 met the inclusion criteria for the study, while 149 were excluded for the presence of DUAOs, clinically significant cardiac arrhythmias or valvular regurgitations, rhabdomyolysis, or lameness detected either during clinical examination or the treadmill tests. The study population consisted of 27 mares, 44 stallions, and 10 geldings aged from 2 to 8 years old (median 3, IQR 3–4) and weighing from 372 to 530 kg (mean 453 ± 34.41 kg).

Twenty-six horses were EIPH-negative at tracheoscopy (32.1%), while the remaining 55 (67.9%) were EIPH-positive; 19 horses had a TBS of 1 (23.46%), 23 had a TBS of 2 (28.39%), 11 had a TBS of 3 (13.58%), and 2 had a TBS of 4 (2.47%). The sTHS ranged from 0 to 272 (median 34, IQR 12–68). The results of the BALf leukocyte differential count in the enrolled horses and in the horses included in the Neu5 group (*n* = 28) are displayed in Table 1.

Concerning the fitness parameters, the median VLa4 value was 8.6 m/s (IQR 7.6–9.3 m/s), the median HRLa4 was 208 bpm (IQR 199.6–214 bpm), and the median V200 was 8 m/s (IQR 7–8.5 m/s). The horses reached a mean peak lactate concentration of 20.5 ± 7.28 mmol/L and a median V max of 11 m/s (IQR 11-11). The values of pH min were measured in 48 horses, while those of Ht max in 31 horses; the average pH min was 7.152 ± 0.099 and the mean Ht max was 64.13 ± 4.13%.

### 3.2. Age, Sex, and Weight

Age was positively correlated with TBS (all horses: *p* = 0.0038, r = 0.32; Neu5: *p* = 0.0002, r = 0.65), sTHS (*p* = 0.0269, r = 0.25), and V max (all horses: *p* = 0.0013, r = 0.35; Neu5: *p* = 0.0017, r = 0.58); in contrast, it was inversely correlated with the BALf eosinophils count (*p* = 0.0104, r = -0.28). When considering only the Neu5 group, no association was found between age and sTHS.

Regarding the sex, geldings were older than mares (*p* = 0.0113), while no differences concerning age were observed when considering males vs. females. Females had significantly higher BALf macrophage counts compared to males (*p* = 0.0106) and lower BALf lymphocytes (*p* = 0.0285) and mast cells (*p* = 0.0308). The BALf eosinophils percentages were higher in stallions compared to geldings (*p* = 0.0191). Males showed significantly higher values of VLa4 (*p* = 0.0037), HRLa4 (*p* = 0.0013) and V200 (*p* = 0.047) compared to females. No association between sex and the EIPH parameters was observed.

Weight was positively correlated with V200 (*p* = 0.0126, r = 0.28; Neu5: *p* = 0.0098, r = 0.48); when considering only the Neu5 group, weight was also correlated with sTHS (*p* = 0.0472, r = 0.38) and VLa4 (*p* = 0.0024, r = 0.55).

### 3.3. EIPH-Related Parameters

**Figure 1:**
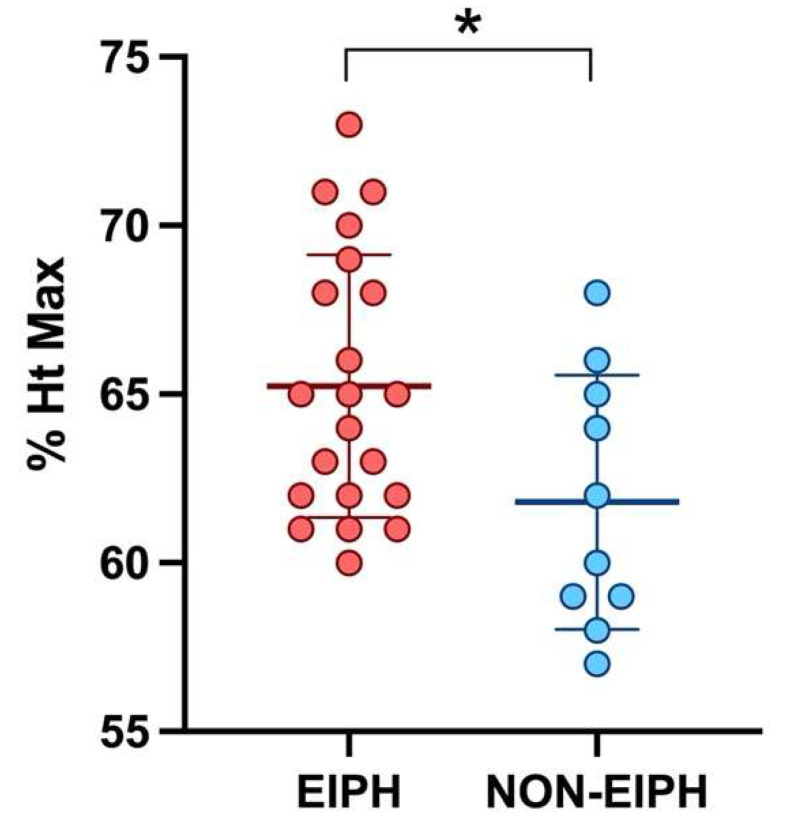
Scatter plot showing the mean and standard deviation of the maximum hematocrit reached during exercise in EIPH and non-EIPH horses. The statistical significance is shown as * (*p* < 0.05).

The TBS was positively correlated with the sTHS (*p* = 0.0430, r = 0.23); moreover, a positive correlation was observed between the TBS and the Ht max (*p* = 0.0072, r = 0.47). When considering only the Neu5 group, a statistical correlation was found between the TBS and the V max (*p* = 0.0323, r = 0.41). Furthermore, horses showing to be EIPH-positive at the tracheobronchoscopy had slightly higher BALf mast cell counts compared to EIPH-negative horses (*p* = 0.0489) and reached higher values of Ht max (*p* = 0.0276) (Figure 1). The sTHS was positively correlated with the BALf mast cells count (*p* = 0.0392, r = 0.23; Neu5: *p* = 0.0024, r = 0.55) and, only in the Neu5 group, with the pH min value (*p* = 0.0488, r = 0.65).

### 3.4. BALf Leukocyte Differential Cell Count

The percentage of BALf macrophages was inversely correlated with the values of VLa4 (*p* = 0.0026, r = −0.33) and HRLa4 (*p* = 0.0006, r = −0.37). The lymphocytes count was positively correlated with VLa4 (*p* = 0.0017, r = 0.34), HRLa4 (*p* = 0.0242, r = 0.25) and V200 (*p* = 0.0342, r = 0.24). The count of neutrophils was inversely correlated with VLa4 (*p* = 0.0035, r = −0.32) and V200 (*p* = 0.0145, r = −0.27), and positively correlated with peak lactate (*p* = 0.0027, r = 0.33). No associations were observed between the eosinophils and mast cells counts and any fitness parameters.

## 4. Discussion

Since the influence of EIPH on performance in racehorses has been widely discussed without reaching univocal results [5,6,7,8,9,10,11,12,13,14,15,16], the present study aimed to objectively define whether EIPH affected some fitness parameters in Standardbreds based on an incremental exercise test on a treadmill. Although our study found no association between EIPH and the main treadmill parameters, the causative role of EIPH in poor racing performance cannot be excluded, as it may be related to different mechanisms. Moreover, higher values of maximum hematocrit reached during exercise were observed in EIPH-affected horses, hinting that the hemoconcentration may contribute to the increase in pulmonary capillary pressure.

Among the population included in the current study, post-exercise tracheobronchoscopy revealed the presence of EIPH in 68% of the horses, which reflects the previously reported prevalence in racehorses [2]. However, the prevalence detected in the present work may be underestimated, as exercise on a treadmill does not accurately resemble what happens during a race. In our study, a significant correlation was observed between the tracheal blood score and the simplified total hemosiderin score, although a single tracheobronchoscopic evaluation was performed. This finding suggests that the EIPH episodes detected at endoscopy did not represent single and isolated events, but EIPH-positive horses were prone to develop repeated episodes of EIPH.

In the present study, both the tracheal blood score and total hemosiderin score were associated with increasing age: in fact, EIPH is a progressive condition [1], which has been associated with the number of racings starts [38] and, consequently, with age [11,13,14]. Moreover, a correlation between age and the maximum speed reached during the treadmill test was observed; it has been reported that trotters have their fastest racing times at about 6 years of age [39,40]. This could be explained by the fact that adult horses are more trained, fit, and physically mature compared to two-year-old horses; furthermore, it is reasonable that only the best-performing horses are selected to keep racing at older ages, while, among novice young racehorses, there may be a wider range of performance quality. Age was also inversely correlated with BALf eosinophilia, in accordance with previous studies [31,41,42]. Males had higher values of VLa4, HRLa4, and V200 compared to females, suggesting that sex had an influence on fitness; an association between sex and VLa4 has already been reported [43], while another study failed to detect any relationship, probably due to the small number of horses included [32]. Moreover, it has been reported that male racehorses are one second faster over one mile [36] and are 1.6 times more likely to win or place than females [44]. In our study, weight was also correlated with VLa4 and V200; it could be hypothesized that bigger horses have a longer stride, making less effort to maintain higher speeds by influencing the economy of locomotion. Moreover, weight was correlated with the simplified total hemosiderin score, suggesting that heavier horses are more prone to suffer from EIPH; to the authors’ knowledge, an association between weight and EIPH has not been previously reported. The hypothesis that EIPH may result from locomotory impact-induced trauma [45,46] could explain this finding; in heavier horses, the impact pressure would be higher, and the resulting shear waves may damage the lung parenchyma more easily. However, the exact pathogenetic mechanisms of EIPH have not been identified yet, and this theory has not been widely accepted [47].

In the current study, all horses presented a BALf cytological profile typical of mild–moderate equine asthma (MEA) [37]; this could be due to the fact that the population included in the study consisted mainly of young racehorses in training, among which MEA can have a prevalence higher than 80% [48]. Since MEA, and, in particular, BALf neutrophilia, has been associated with poor performance [48,49,50,51], the lack of non-asthmatic horses may represent a limitation to this study. When more than one poor-performance-associated disease is observed in the same subject, the real cause of the lower athletic capacity can be debatable, as it may result from the combination of different factors. Also, in the present study, higher percentages of neutrophils were correlated with lower VLa4 and V200, and with higher peak lactate concentration, confirming the relationship between neutrophilic lung inflammation and lower athletic capacity observed in a previous study [32]. Therefore, among our study population, we selected a group of horses showing a neutrophils percentage of < 5% in order to evaluate the influence of EIPH alone on the fitness parameters, in particular, those associated with neutrophilia (VLa4, V200, and peak lactate). Nevertheless, no association between EIPH and V200, VLa4, HRLa4, and peak lactate was detected, even in the Neu5 group. Similarly, previous studies including a small number of horses failed to observe any relationship between the presence of EIPH and blood lactate values [52,53]; only one study identified higher peak lactate after exercise in EIPH-affected horses [54]. Moreover, no difference in the neutrophil percentage between the EIPH-positive and negative groups was detected, and no association between neutrophils count and sTHS was observed. This result suggests that EIPH does not directly influence the fitness capacity of racehorses, which depends on multiple variables. However, in our study, the maximal oxygen consumption (VO_2max_), expressing the maximal aerobic capacity and reflecting athletic performance [17], was not measured; future studies may be conducted to investigate a possible association between EIPH and VO_2max_. Finally, a possible role of EIPH in the racing performance cannot be ruled out; this should not be sought in decreased fitness, but it should be reasonably investigated in a different way. Interestingly, horses with higher percentages of mast cells had higher tracheal blood scores and simplified total hemosiderin scores, suggesting an association between BALf mastocytosis and EIPH; similar results had been reported by a previous study, where the hemosiderophage counts were higher in horses affected by mastocytic MEA compared to those with neutrophilic MEA [55].

In our study, the tracheal blood score, observed at endoscopy after treadmill exercise, was correlated with higher speeds reached during the treadmill test; it has been reported that the risk of EIPH is higher for sprinter horses racing 1000–1200 m compared with horses racing longer distances [37], and that epistaxis is more common after races < 1600 m than in longer races [56]. However, different studies detected no direct association between race speed and EIPH [5,37], while a faster average early/mid-race speed has been associated with EIPH scores of ≥ 3 [5]; this could be explained by the fact that rapid acceleration triggers higher pulmonary vascular pressures than a gradual incremental increase to the same speed [57]. Also, a study reported that barrel racing horses with the most severe grade of EIPH were faster than the EIPH-negative ones [58], confirming rapid acceleration as a risk factor for EIPH.

Moreover, EIPH-positive horses reached, in the present study, higher values of Ht max during exercise—the more the hematocrit raises, the more the oxygen-carrying capacity of the horse’s blood during exercise increases; however, no association has been reported between post-exercise Ht and performance [26]. To explain the association between EIPH and Ht, it should be considered that higher values of Ht have been related to a higher mean arterial pressure during treadmill exercise in horses [59] and, in human medicine, red blood cell aggregation seems to participate in the increase of pulmonary capillary pressure [60]; therefore, a hypothetical explanation may be that hemoconcentration might play a role in the pathogenesis of pulmonary hemorrhage in horses. Moreover, it could be hypothesized that higher values of Ht max could provide a more efficient buffering capacity of the acid–base disturbance during exercise [61] in EIPH-positive horses; interestingly, in our study, the simplified total hemosiderin score was associated with higher values of pH min reached during the treadmill test, and, therefore, a lower grade of exercise-induced acidosis, supporting this hypothesis. However, as hematocrit variations during exercise are influenced by different factors, such as the splenic reserve and the intercompartmental fluid shifts, further studies are needed to investigate the relationship and the possible pathogenetic role of Ht in EIPH.

## 5. Conclusions

The present study showed no influence of EIPH on treadmill parameters, such as VLA4, HRLa4, and V200, suggesting that EIPH does not impair fitness in Standardbred racehorses; however, this finding does not rule out the causative role of EIPH in decreased racing performance, which should reasonably be further investigated. Interestingly, horses affected by EIPH reached higher values of hematocrit, suggesting a possible role of hemoconcentration in the increase of pulmonary capillary pressure.

## Figures and Tables

**Table 1:** Leukocyte differential counts of the bronchoalveolar lavage fluids of all horses and those included in the Neu5 Group. Data are expressed as mean ± standard deviation if normally distributed, or median (IQR) if not normally distributed.

Cell Population	All Horses	Neu5 Group
Macrophages	43.33% ± 10.66%	39.46% ± 10.59%
Lymphocytes	36.43% ± 13.71%	46% (38.25–52.75%)
Neutrophils	9% (5–17%)	3% (2.25–5%)
Eosinophils	1% (0–3%)	1.5% (0–6%)
Mast cells	4% (3–6%)	5% (4–7%)

## Data Availability

The data presented in this study are available on request from the corresponding author.
